# Identifying acute myeloid leukemia subtypes based on pathway enrichment

**DOI:** 10.3389/fphar.2025.1557112

**Published:** 2025-03-21

**Authors:** Ling Zhong, Jiangti Luo, Junze Dong, Xiang Yang, Xiaosheng Wang

**Affiliations:** ^1^ Biomedical Informatics Research Lab, School of Basic Medicine and Clinical Pharmacy, China Pharmaceutical University, Nanjing, China; ^2^ Intelligent Pharmacy Interdisciplinary Research Center, China Pharmaceutical University, Nanjing, China; ^3^ Big Data Research Institute, China Pharmaceutical University, Nanjing, China; ^4^ Institute of Innovative Drug Discovery and Development, China Pharmaceutical University, Nanjing, China; ^5^ Nanjing Foreign Language School, Nanjing, China; ^6^ Department of Oncology, JunXie Hospital, Nanjing, China

**Keywords:** AML, acute myeloid leukemia, pathway enrichment analysis, transcriptome (RNA-seq), drug sensitivity, subtype

## Abstract

Acute myeloid leukemia (AML) is the most common type of acute leukemia in adults and the second most common in children. Despite the introduction of targeted therapies, AML survival rates have shown limited improvement, particularly among older patients. This study explored personalized treatment strategies for AML by proposing a novel subtyping method. Through unsupervised clustering based on the enrichment scores of 14 pathways related to metabolism, immunity, DNA repair, and oncogenic signaling, we identified three AML subtypes: DNA repair (DR), immune-enriched (ImE), and immune-deprived (ImD), consistent in four independent datasets. DR is marked by high expression of DNA repair and metabolic pathways, high stemness and proliferation potential, as well as high sensitivity to chemotherapy. ImD is characterized by low expression of immune and oncogenic pathways, favorable survival prognosis, low mutation rates of *RUNX1* and *TP53*, high homeostasis, and low migration potential. ImE exhibits high enrichment of immune and oncogenic pathways, low stemness and proliferation capacity, low homeostasis, high migration potential, and low sensitivity to chemotherapy. Our pathway enrichment-based subtyping approach would offer a promising framework for understanding the molecular heterogeneity of AML and guiding personalized treatment of this disease.

## Introduction

Acute myeloid leukemia (AML) is the most common type of acute leukemia in adults and the second most common in children (Carter et al., 2020). This disease is marked by a block in cellular differentiation and the clonal proliferation of abnormal myeloblasts within the bone marrow. AML is endowed with a highly heterogeneous clinical course, with diverse molecular features playing crucial roles in risk assessment, prognosis, and treatment selection (Döhner et al., 2017; Döhner et al., 2015; Arber et al., 2016; Papaemmanuil et al., 2016a). In recent decades, the classification of AML has evolved from the French-American-British (FAB) morphological subtyping to the more refined World Health Organization (WHO) system (Khoury et al., 2022). More recently, with the advancement of high-throughput technologies, genomics and transcriptomics-based classification of AML has been proposed. For instance, The Cancer Genome Atlas (TCGA) program has dissected the genomic landscape of AML, identifying nine categories of gene mutations (null et al., 2013). Another landmark study has established 11 distinct AML classes based solely on genetic abnormalities, whereas 4% of AML patients met the criteria for two or more classes, 11% remained unclassified, and 5% showed no driver mutations (Papaemmanuil et al., 2016b).

Despite these advances in AML subtyping, the survival rate of this disease has achieved modest improvement over the past few decades. One main reason for the limited treatment improvement is the continued use of the “7 + 3” standard induction regimen, a standard treatment option, particularly for younger patients (Döhner et al., 2015), consisting of the chemotherapeutic agents cytarabine (Ara-C) and daunorubicin. The limited effectiveness of this treatment, especially in older populations, can be attributed to the high toxicity of these drugs and the heterogeneity of this disease (Eleni et al., 2010; Hassan et al., 2017). New insights into the biology of AML have revealed increasingly apparent AML heterogeneity (Lohse et al., 2018). To improve the treatment effectiveness for AML, a number of targeted therapies have recently been investigated in clinical trials, particularly those targeting AML cell survival pathways (Carter et al., 2020), including the FDA-approved agents midostaurin (*FLT3* inhibitor) (Novartis Pharmaceuticals Corp, 2017), gemtuzumab ozogamicin (anti-*CD33* antibody-drug conjugate) (Appelbaum and Bernstein, 2017), CPX-351 (liposomal cytarabine/daunorubicin) (Feldman et al., 2011), enasidenib (Celgene Corporation, 2017) and ivosidenib (Agios Pharmaceuticals, 2018) (*IDH1/2* inhibitors), gilteritinib (*FLT3* inhibitor) (Astellas Pharma, 2018), venetoclax (*BCL-2* inhibitor) (AbbVie Inc. and Genentech Inc, 2020), and glasdegib (Hedgehog pathway inhibitor) (Pfizer, 2018). Consequently, there is a shift from a “one-size-fits-all” approach towards more targeted personalized therapies (Lohse et al., 2018).

To explore personalized targeted therapeutics for AML, we proposed a new subtyping method for AML by unsupervised clustering based on pathway enrichment scores. As the pathway enrichment score integrates the expression levels of multiple genes into a single value, the pathway enrichment-based clustering may result in more stable and robust results than the gene expression-based clustering. Moreover, the former can generate more straightforward and explainable results for cancer subtypes than the latter. Here we used 14 cancer-associated pathways to cluster AML specimens. These pathways were involved in metabolism (glycolysis/gluconeogenesis), immunity (natural killer cell-mediated cytotoxicity, antigen processing and presentation, T cell receptor signaling, B cell receptor signaling, and JAK-STAT signaling), DNA repair (cell cycle, mismatch repair, and homologous recombination), and oncogenic signaling (PI3K/Akt, TGF-*β*, Wnt, Hedgehog, and mTOR). The pathway enrichment-based clustering analysis identified three subtypes of AML, consistent in three independent datasets. We further comprehensively compared molecular and clinical characteristics among these AML subtypes. Furthermore, we employed this method in an AML single cell RNA-Seq (scRNA-seq) dataset to exhibit its generality.

## Methods

### Datasets

From the Genomic Data Commons Data Portal (https://portal.gdc.cancer.gov/), we downloaded multi-omics datasets and clinical data for the TCGA AML cohort (termed TCGA-LAML), consisting of 173 adult AML patients. The TCGA-LAML multi-omics datasets included gene expression profiles (RSEM-normalized), and somatic mutation data (MAF files). From *cBioPortal* for Cancer Genomics (https://www.cbioportal.org/), we downloaded transcriptomic and clinical datasets for another AML cohort (Beat-AML (Tyner et al., 2018)), consisting of 143 adult AML patients. From the UCSC database (https://xenabrowser.net/datapages/), we downloaded transcriptomic and clinical datasets for a pediatric AML cohort (TARGET-AML (Gamis et al., 2014; Aplenc et al., 2008; Aplenc et al., 2020)), consisting of 561 samples with survival information. In addition, we obtained transcriptomic and clinical datasets for two AML cohorts (GSE106291 (Tobias et al., 2018) and GSE71014 (Chuang et al., 2015)) from the NCBI Gene Expression Omnibus (GEO) (https://www.ncbi.nlm.nih.gov/geo/). The GSE106291 and GSE71014 datasets contained 250 and 104 adult AML patients, respectively. From GEO, we also downloaded an AML scRNA-seq dataset (GSE116256 (van Galen et al., 2019)).

### Gene-set enrichment analysis

To systematically investigate the impact of targeted therapies on acute myeloid leukemia (AML) through survival pathway modulation, we curated and compiled 14 signaling pathways from the KEGG database into gene sets. These pathways were systematically classified into four functional categories: metabolism (glycolysis/gluconeogenesis (Yang et al., 2024)), immunity (natural killer cell-mediated cytotoxicity (Merino et al., 2023), antigen processing and presentation, T cell receptor signaling (Wang et al., 2024), B cell receptor signaling (Guo et al., 2024), and JAK-STAT signaling (Venugopal et al., 2020; Moser et al., 2021)), DNA repair (cell cycle, mismatch repair, and homologous recombination), and oncogenic signaling (PI3K/Akt (Nepstad et al., 2020; Darici et al., 2020), TGF-*β* (Naji et al., 2024; Dong and Blobe, 2006), Wnt (Láinez-González et al., 2023), Hedgehog (Shallis et al., 2019; Terao and Minami, 2019; Hoy, 2019), and mTOR (Nepstad et al., 2020; Darici et al., 2020)). We determined the enrichment score of a gene set in a tumor sample using the single-sample gene set enrichment analysis (ssGSEA) (Hänzelmann et al., 2013). We employed the ssGSEA algorithm implemented in the *GSVA* R package, to comprehensively assess pathway enrichment for each sample in this study. A gene set is composed of the genes in a pathway or marker genes of a specific biological process (stemness, proliferation score, migration, and homeostasis) or signature. The gene sets we analyzed are presented in Supplementary Table S1.

### Determination and analysis of deregulated genes in pathways

Differential analysis of the 14 pathways in the AML expression data from the TCGA cohort was performed using the *limma* package, based on the pathway-based subtypes identified in our study. In order to select differentially expressed genes (DEGs), specific selection criteria were applied, requiring an adjusted p-value of less than 0.05 and an absolute log-fold change of greater than 1.5. We constructed a protein-protein interactions (PPI) network of the overlapping genes using String (https://string-db.org/) (Szklarczyk et al., 2019) with “*Homo sapiens*” as the selected species and the minimum required interaction score set to the highest confidence (0.7). The resulting “TSV” file was imported into Cytoscape (https://cytoscape.org/) (Shannon et al., 2003) for network analysis and visualization with the isolated proteins excluded. The degree of the PPI network were obtained by running the “NetworkAnalyzer ()” function.

### Clustering analysis

We employed the hierarchical clustering algorithm to identify AML subtypes based on the enrichment levels (ssGSEA scores) of the 14 pathways. The clustering analysis was performed with the R package *hclust*. To determine the optimal number of clusters, we systematically evaluated hierarchical clustering results as follows: The dendrogram derived from hierarchical clustering was cut at varying heights to generate different numbers of clusters. For each candidate cluster number (*k* = 3–10), we computed the average silhouette coefficient across all samples, which quantifies both intra-cluster cohesion and inter-cluster separation. The optimal number of clusters was selected as the value of k that maximized the average silhouette coefficient, ensuring robust and biologically meaningful subtype definitions (Supplementary Figure S1).

### Drug correlation evaluation

We utilized the tool *OncoPredits* (Maeser et al., 2021) for drug sensitivity analysis, which predicts drug responses based on baseline transcriptomic data of cell lines. Using the *limma* R package, we performed differential analysis of drug sensitivity to identify drugs having significant differences in sensitivity across AML subtypes.

### Gene coexpression network analysis

We applied the weighted gene coexpression network analysis (WGCNA) algorithm (Langfelder and Horvath, 2008) to identify gene modules significantly correlated with a specific trait, with the gene expression matrix and sample labels as input. By analyzing the expression correlation between hub genes in gene modules, we determined associated gene ontology (GO) terms. The WGCNA analysis was performed using the R package *WGCNA* (version 1.73).

### Analysis of scRNA-seq data

The scRNA-seq dataset GSE116256 was gene expression profiles in 15,675 single cells from 16 untreated AML patients, consisting of 9,590 malignant cells and 6,085 non-malignant cells. For the scRNA-seq dataset, we performed quality control according to guide in the original publication (van Galen et al., 2019). We used the Uniform Manifold Approximation and Projection (UMAP) algorithm (McInnes et al., 2018) to visualize malignant cells and non-malignant cells, respectively.

### Survival analysis

We compared overall survival rates among different groups of cancer patients using the Kaplan-Meier (K-M) method (Bland and Altman, 1998). The significance of differences in survival rates was assessed using the log-rank test. We performed survival analysis using the survfit () function in the R package *survival* (version 3.5.5).

### Statistical analysis

To compare two classes of normally distributed data, such as gene expression levels, we used the two-tailed Student’s t-test. For comparing two classes of data that were not normally distributed, we employed the two-tailed Mann-Whitney *U* test. When comparing three classes of data, if they were normally distributed, we used the one-way ANOVA; otherwise, we used the Kruskal–Wallis (K-W) test. For analyzing contingency tables, we employed the Fisher’s exact test. To adjust for *P* values in multiple testing, we used the Benjamini–Hochberg method (Benjamini and Hochberg, 1995) to calculate the false discovery rate (FDR). All statistical analyses were conducted using the R programming language (version 4.3.1).

## Results

### Pathway enrichment-based clustering identifies three AML subtypes

**FIGURE 1:**
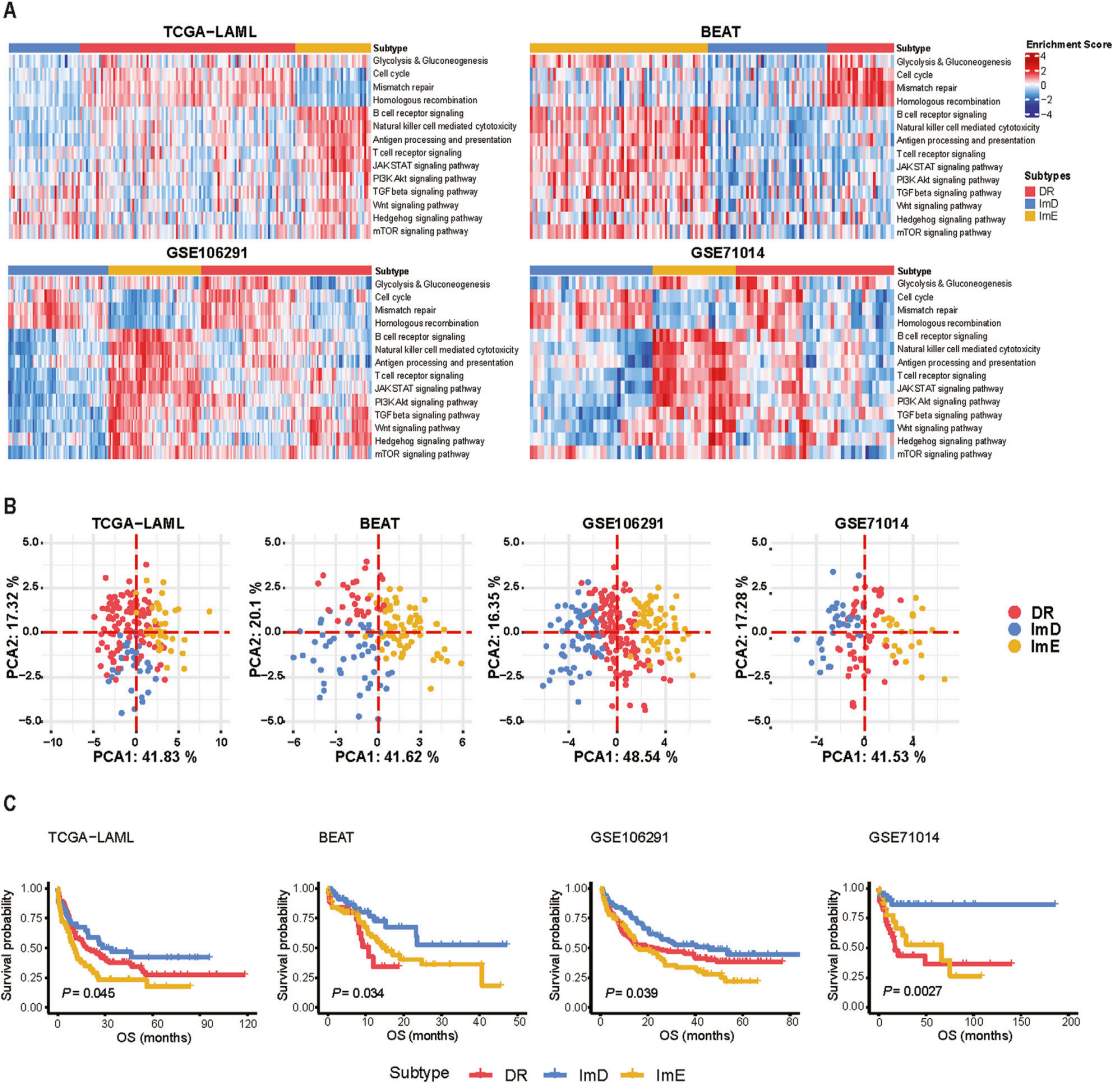
Hierarchical clustering identifies three AML subtypes based on the enrichment scores of 14 pathways. **(A)** Heatmap showing three AML subtypes: DR, ImE, and ImD, identified based on the enrichment scores of 14 pathways in four different datasets (TCGA-LAML, BEAT-AML, GSE106291, and GSE71014). The pathway enrichment scores were calculated by the ssGSEA algorithm. The 14 pathways are involved in metabolism, immune, DNA repair, and oncogenic signaling. **(B)** PCA confirms that AML can be clearly separated into three subgroups based on the ssGSEA scores of the 14 pathways. **(C)** Kaplan-Meier curves show that ImD tends to have the best survival prognosis among the three subtypes. The log-rank test *P* values are shown. OS: overall survival.

Based on the pathway enrichment scores (ssGSEA scores) of the 14 pathways related to metabolism, immune, DNA repair, and oncogenic signaling, we uncovered three AML subtypes by hierarchical clustering, consistent in four independent AML datasets (TCGA-LAML (Ley et al., 2013), BEAT-AML (Tyner et al., 2018), GSE106291 (Tobias et al., 2018), and GSE71014 (Chuang et al., 2015)). By integrating the average silhouette coefficient and identifying natural cut points in the hierarchical clustering dendrogram, we determined the optimal number of clusters to be *k* = 3 (Supplementary Figure S1). In terms of pathway enrichment levels, we termed the three subtypes DNA repair (DR), immune-enriched (ImE), and immune-deprived (ImD), respectively (Figure 1A). The DR subtype was characterized by hyperactivation of glycolysis/gluconeogenesis, DNA repair, and cell cycle pathways; the ImE subtype showed high enrichment in immune and oncogenic pathways; and the ImD subtype exhibited low expression of immune and oncogenic pathways. Principal component analysis supported that AML can be clearly separated into these three subtypes based on the pathway enrichment scores (Figure 1B). Survival analysis revealed that the ImD subtype had the highest overall survival rates compared to the other subtypes (log-rank test, *P* < 0.05) (Figure 1C). In parallel, we applied a pathway-based clustering approach to analyze the pediatric acute myeloid leukemia (AML) dataset. By examining pathway enrichment scores, we identified a clustering pattern that resembled the classification observed in adult AML, with the data being similarly subdivided into three distinct subtypes (Supplementary Figures S2A, B). However, despite these clustering similarities, survival analysis did not reveal any significant prognostic differences across the three subtypes (Supplementary Figure S2C). Since the ImD subtype has the highest survival rate, we performed differential gene expression analysis between the ImD subtype and the remaining samples. In the 14 pathways, we identified a total of 10 upregulated genes and 50 downregulated genes (Supplementary Figure S3A, Supplementary Table S2). The PPI network of the 60 deregulated genes was constructed by STRING. The network contained 46 nodes and 232 edges with an average node degree of 10.1, which was further analyzed and visualized using Cytoscape [32] (Supplementary Figure S3B). In the PPI network, nine top potential targets (CD4, IL10, FCER1G, FCGR3B, FCGR3A, TLR2, LILRB2, TLR4, ITGB2) were selected according to the Degree ranking (Supplementary Tables S3, 4).

### The AML subtypes display different pathway and biological process enrichment

**FIGURE 2:**
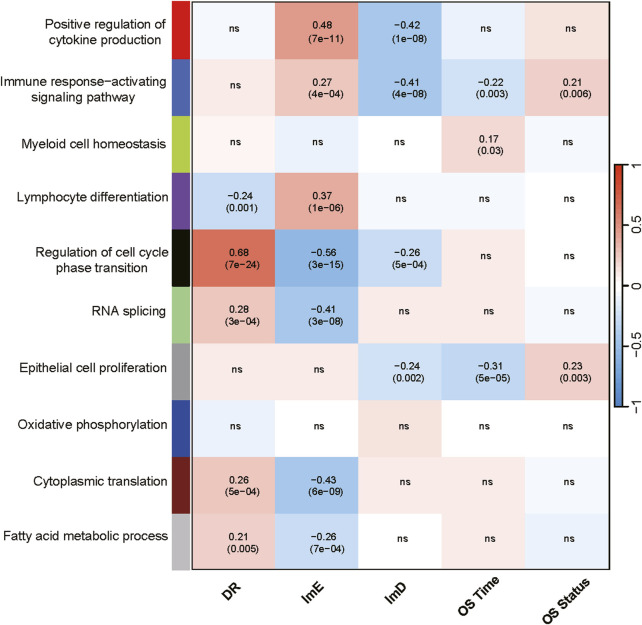
Gene modules and their representative GO terms significantly differentiating AML by the subtypes in TCGA-LAML. The correlation coefficients and *P* values (in parenthesis) generated by WGCNA are shown. OS, overall survival.

In the TCGA-LAML dataset, we identified nine gene modules by WGCNA (Langfelder and Horvath, 2008), whose enrichment showed significant correlations with the AML subtypes, overall survial time and/or overall survival status (Figure 2). The representative GO biological processes for these modules included positive regulation of cytokine production, immune response-activating signaling pathway, myeloid cell homeostasis, lymphocyte differentiation, regulation of cell cycle phase transition, RNA splicing, epithelial cell proliferation, cytoplasmic translation, and fatty acid metabolic process (Figure 2). As expected, three immune-related modules (positive regulation of cytokine production, immune response-activating signaling pathway, lymphocyte differentiation) had significant, positive correlations with the ImE subtype, while they showed negative correlations with ImD or DR subtypes (*P* < 0.05, |correlation coefficient| > 0.2). Of the immune-related modules, the immune response-activating signaling pathway correlated negatively with overall survival time (*P* = 0.003, |correlation coefficient| > 0.2). Moreover, the myeloid cell homeostasis module correlated positively with overall survival time (*P* = 0.03, correlation coefficient = 0.17). This is justified as AML is an immune cell proliferation-associated cancer. We also observed four modules (regulation of cell cycle phase transition, RNA splicing, cytoplasmic translation, and fatty acid metabolic process) showing significant, positive correlations with the DR subtype but negative correlations with ImE or ImD subtypes (*P* < 0.05, |correlation coefficient| > 0.2) (Figure 2). It aligns with the high enrichment of metabolism, DNA repair, and cell cycle pathways in DR. In addition, the epithelial cell proliferation module had a significant, negative correlation with the ImD subtype as well as overall survival time (*P* < 0.05, correlation coefficient < −0.2) (Figure 2). Again, it aligns with the property of ImD that is characterized by low enrichment of immune pathways and favorable survival prognosis.

### The AML subtypes display different mutation profiles

**FIGURE 3:**
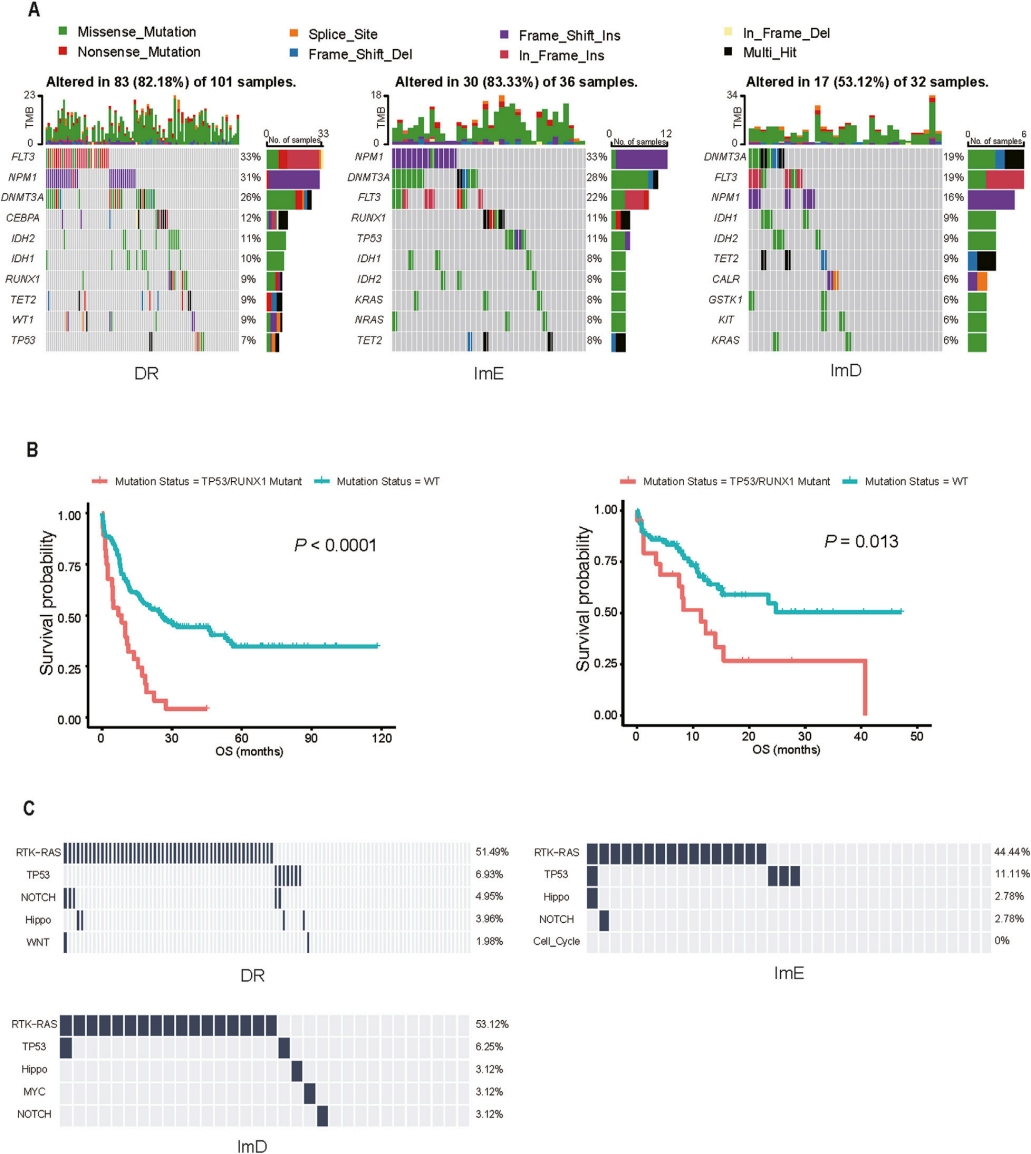
Comparisons of mutation profiles among the AML subtypes in TCGA-LAML. **(A)** Top 10 genes with the highest mutation rates in the three subtypes. **(B)** Kaplan-Meier curves showing significantly poorer overall survival rates in the *TP53* or *RUNX1* mutant groups compared to their wild-type counterparts. **(C)** Oncogenic pathways associated with the frequently mutated genes in the three subtypes. The bars indicate the proportions of samples with the pathway genes mutated.

AML genomes have fewer mutations than most other adult cancers, with an average of 13 mutations found in genes (null et al., 2013). We compared the mutational landscape among the AML subtypes in the TCGA-LAML cohort. Figure 3A shows the top 10 genes with the highest mutation frequencies in each subtype. Notably, six genes (*FLT3*, *NPM1*, *DNMT3A*, *IDH2*, *IDH1*, and *TET2*) were included in the top 10 most frequently mutated genes in all three subtypes, and *FLT3*, *NPM1*, and *DNMT3A* were identified as three most frequently mutated genes in all three subtypes. We found that *RUNX1* and *TP53* had higher mutation rates in ImE than in DR and ImD (*RUNX1*: 11% (ImE) vs 9% (DR) vs 6% (ImD); *TP53*: 11% (ImE) vs 7% (DR) vs 6% (ImD)). To further explore the clinical implications of *TP53* and *RUNX1* mutations, we stratified patients from the TCGA-LAML and Beat-AML datasets into *TP53*/*RUNX1* mutant and wild-type groups. Kaplan-Meier survival analysis demonstrated significantly poorer overall survival in the *TP53* or *RUNX1* mutant groups compared to their wild-type counterparts (*P* < 0.05) (Figure 3B). It aligns with the previous result that the ImD subtype has the best overall survival among the AML subtypes. In addition, we used the *pathways* function from the *maftools* (Mayakonda et al., 2018) package to check for enrichment of known oncogenic signaling pathways (Sanchez-Vega et al., 2018) associated with frequently mutated genes in TCGA-LAML. This analysis revealed that the RTK-RAS pathway was the most prevalent oncogenic pathway mutated across the AML subtypes, being identified in 51%, 53%, and 44% of DR, ImD, and ImE samples, respectively (Figure 3C).

### The AML subtypes display different malignant properties

**FIGURE 4:**
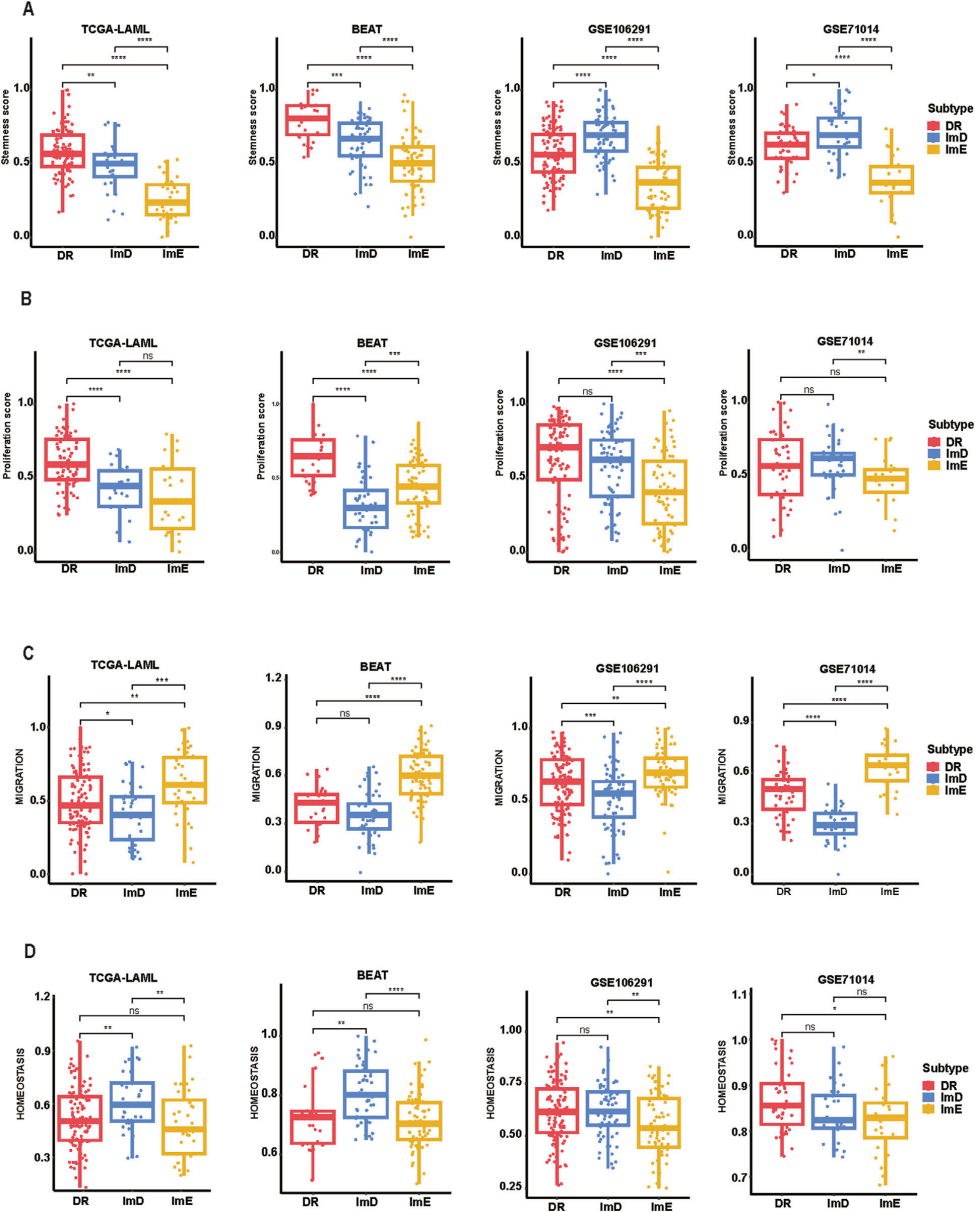
Comparisons of tumor-associated properties among the AML subtypes. Comparisons of stemness **(A)**, proliferation **(B)**, migration **(C)**, and homeostasis scores **(D)** among the AML subtypes. The two-tailed Mann–Whitney *U* test *P* values are shown. *P < 0.05, **P < 0.01, ***P < 0.001, ****P < 0.0001, ns *P* ≥ 0.05.

Certain malignant cells exhibit stem cells-like characteristics, characterized by extremely high proliferation potential (Lei et al., 2024). In the TCGA and BEAT AML datasets, DR exhibited the highest stemness scores, while ImE had the lowest stemness scores (Kruskal–Wallis test, *P* < 0.001) (Figure 4A). Likewise, in the GSE71014 and GSE106291 datasets, ImE displayed the lowest stemness scores (*P* < 0.001). These results suggest that the DR subtype likely has the strongest proliferation potential, while the ImE subtype has the lowest proliferation capacity. Similar results were found in proliferation scores (Figure 4B). We also compared the migration and homeostasis scores among the AML subtypes. In the TCGA, BEAT, and GSE106291 datasets, ImD showed the lowest migration scores, while ImE had the highest scores (Figure 4C). Conversely, the homeostasis scores showed a consistent pattern across all four datasets: ImE exhibited the lowest homeostasis scores, and ImD had the highest (Figure 4D). It suggests that ImE has the highest potential in cell migration, invasion, and metastasis to contribute to disease progression. By contrast, ImD shows the lowest cell migration potential that may lead to its relatively favorable prognosis.

### The AML subtypes show different drug response

**FIGURE 5:**
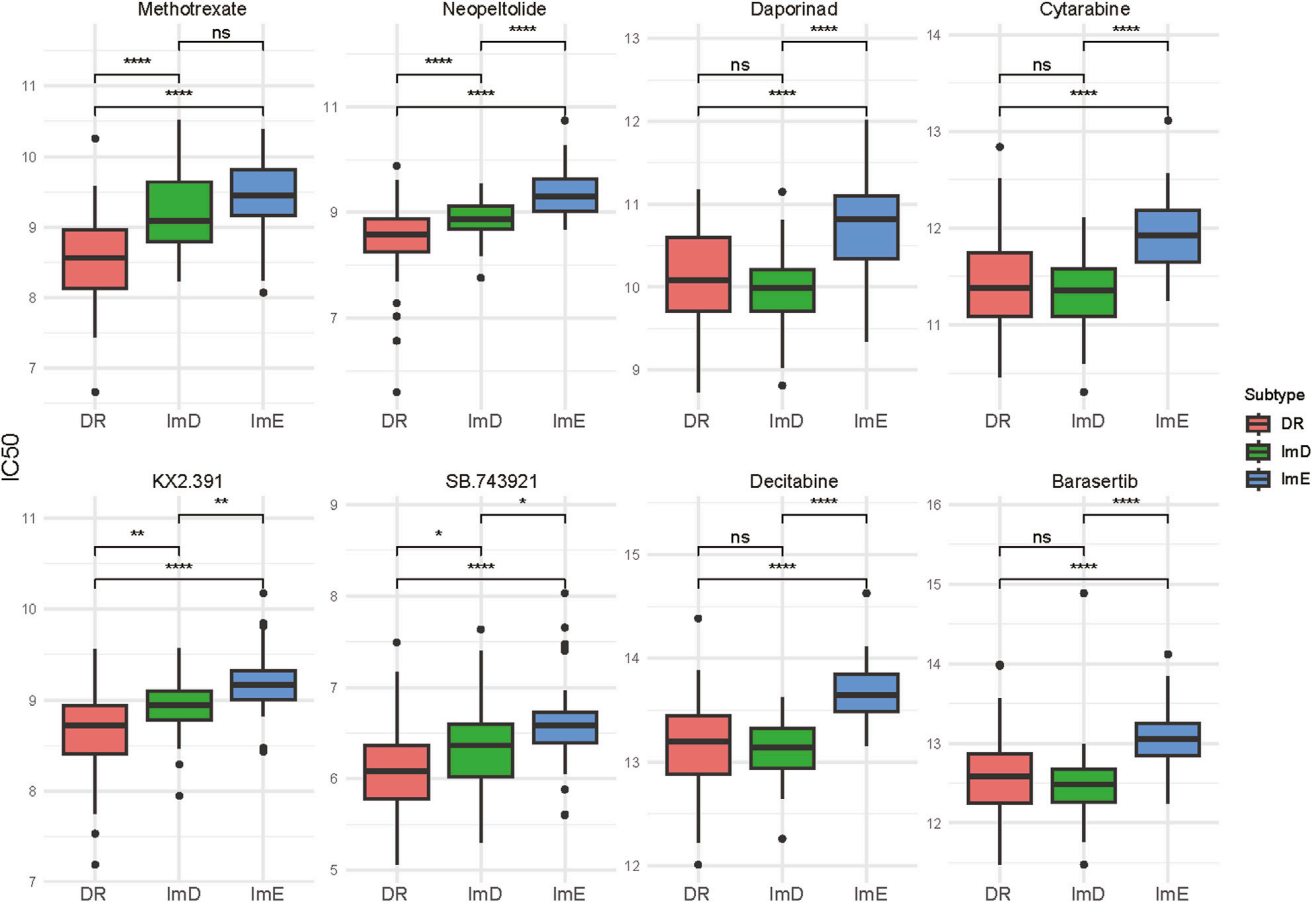
Eight chemotherapy drugs to which the AML subtypes show significantly different sensitivity. The two-tailed Mann–Whitney *U* test *P* values are shown. *P < 0.05, **P < 0.01, ***P < 0.001, ****P < 0.0001, ^ns^ *P* ≥ 0.05.

Drug sensitivity analysis identified eight chemotherapy drugs to which the AML subtypes had significantly different sensitivity. These drugs included methotrexate, neopeltolide, daporinad, decitabine, barasertib, cytarabine, KX2.391, and SB.743921. Among them, KX2.391 is a synthetic, orally bioavailable small molecule inhibitor of Src tyrosine kinase signaling and tubulin polymerization; this compound is distinct from other Src kinase inhibitors by targeting the peptide substrate rather than the ATP binding site, with its novel binding site on hetero-dimeric tubulin being distinct from taxanes and other known tubulin inhibitors53. SB.743921 is a derivative of ispinesib, found to be 5-fold more potent against ATPase activity of, E.g.,5 and currently undergoing phase II clinical trials54. Notably, the ImE subtype consistently exhibited the lowest sensitivity to these drugs (Figure 5). By contrast, the DR subtype displayed the highest sensitivity to four of the eight drugs, including methotrexate, neopeltolide, KX2.391, and SB.743921. These drugs belong to the category of cell cycle/cell structure disruptors, which directly inhibit tumor cell proliferation by blocking DNA synthesis, microtubule dynamics, or mitosis. This suggests that the DR subtype may be particularly sensitive to agents targeting key processes in the cell cycle. Among the drugs, decitabine is suitable for elderly cancer patients who are not candidates for intensive chemotherapy; it also serves as an alternative therapy for patients with refractory or relapsed AML (Heuser et al., 2020). Cytarabine is widely used in newly diagnosed AML patients as part of induction therapy to achieve remission, and used in refractory or relapsed AML patients for salvage therapy (Heuser et al., 2020).

### Validation by analyzing scRNA-seq data

**FIGURE 6:**
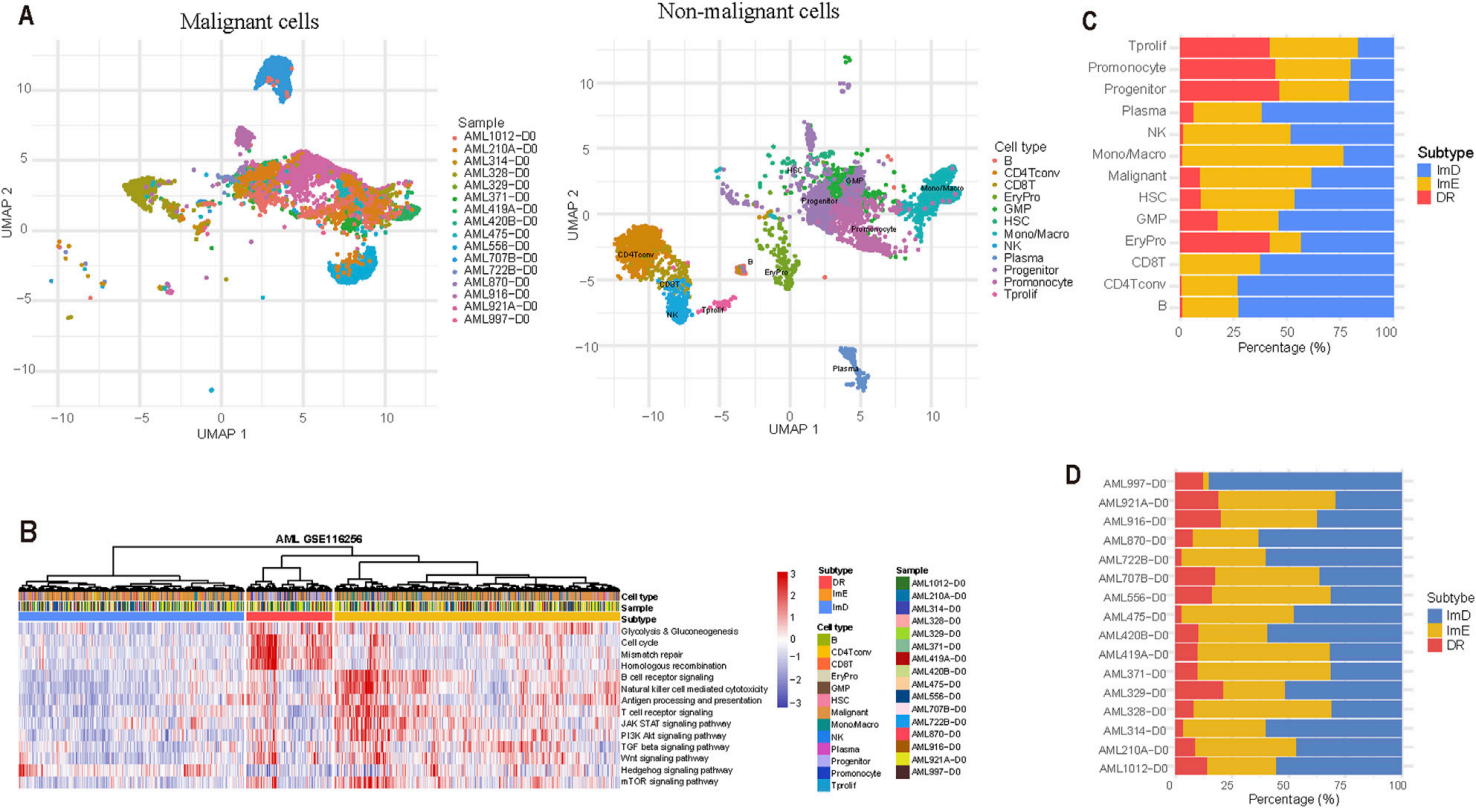
Validation of the pathway-based clustering method in a single-cell RNA-Seq (scRNA-seq) dataset. **(A)** Clustering 9,590 malignant cells and 6,085 non-malignant cells by the UMAP algorithm. **(B)** Hierarchical clustering based on the enrichment scores of the 14 pathways identifies three subtypes of 15,675 single cells from 16 AML patients. **(C)** Distribution of the 15,675 single cells across the three AML subtypes. **(D)** Distribution of the three subtypes in the 16 samples.

We utilized a pathway-based clustering approach to analyze a scRNA-seq dataset (GSE116256). This dataset included the gene expression profiles of 15,675 single cells obtained from 16 patients diagnosed with acute myeloid leukemia (AML). Of these cells, 9,590 were classified as tumor cells, whereas the remaining 6,085 cells represented various immune cell types. Specifically, these immune cells comprised 159 B cells (2.6%), 566 conventional CD4^+^ T cells (CD4 Tconv) (9.3%), 148 CD8^+^ T cells (2.4%), 202 erythroid progenitors (EryPro) (3.3%), 750 granulocyte-monocyte progenitors (GMP) (12.3%), 43 hematopoietic stem cells (HSC) (0.7%), 1,235 monocytes/macrophages (Mono/Macro) (20.3%), 274 natural killer (NK) cells (4.5%), 231 plasma cells (3.8%), 1,653 progenitor cells (27.2%), 788 promonocytes (13.0%), and 36 proliferating T cells (Tprolif) (0.6%). (Figure 6A). We performed hierarchical clustering of the 15,675 single cells based on the enrichment scores of the 14 pathways and identified three subtypes of these cells (Figure 6B). Of the 9,590 malignant cells, 5,011 (52.2%), 3,718 (38.8%), and 861 (9%) cells were classified into ImE, ImD, and DR subtypes, respectively (Figure 6C). The distribution of the three subtypes in the 16 samples is presented (Figure 6D). This result demonstrates the reproducibility of this AML subtyping method at the single-cell level.

### Comparison Between pathway enrichment Subtyping and Other Clinical Subtyping Methods

**FIGURE 7:**
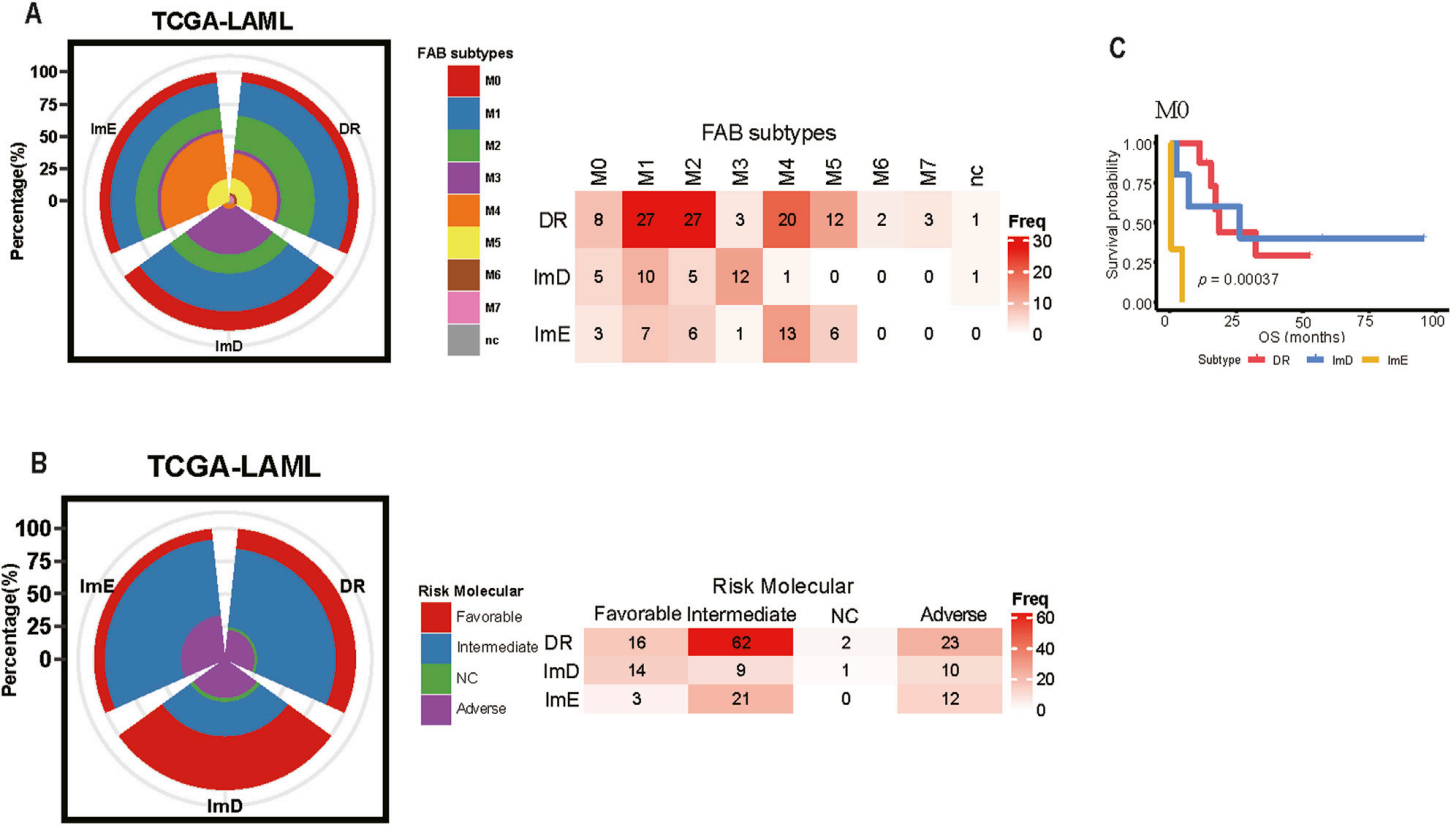
Comparison Between the Pathway enrichment Subtyping and Other Clinical Subtyping Methods in AML. **(A)** Overlapping between the Pathway enrichment Subtyping and FAB Subtyping **(B)** Overlapping between the Pathway enrichment Subtyping and the ELN molecular risk Subtyping **(C)** The cases in the M0 stratified by the Pathway enrichment subtyping show different overall survival prognosis.

Although the FAB classification provides a fundamental framework for diagnosing AML (Bennett et al., 1976), it is noteworthy that most M1 and M2 cases are classified within the DR subtype, while M3 cases predominantly fall under the ImD subtype. M4 and M5 cases are almost entirely categorized within the DR and ImE subtypes, with only one case assigned to the ImD subtype (Fisher’s exact test, *P* < 0.05). Due to the limited sample size, M6 and M7 cases are exclusively found within the DR subtype (Figure 7A). M1 and M2 represent partially undifferentiated leukemias, with M2 specifically associated with the t (8; 21) chromosomal abnormality. This suggests that M1/M2 patients may exhibit significant abnormalities in DNA damage repair mechanisms. M3, classified as acute promyelocytic leukemia (APL), is associated with t (15; 17) and is particularly sensitive to all-trans retinoic acid (ATRA) treatment, with a tendency toward immunodeficiency characteristics. As the understanding of the molecular mechanisms of AML deepens, the ELN molecular risk stratification provides a more precise prognostic assessment (Döhner et al., 2022). Cases classified as “favorable” risk are primarily distributed within the DR and ImD subtypes, while most “intermediate” risk cases fall under the DR subtype. Nearly half of the “adverse” risk cases are also assigned to the DR subtype (Figure 7B). Additionally, M0 cases within the DR, ImD, and ImE subtypes exhibit significantly different overall survival (OS) outcomes (*P* < 0.05) (Figure 7C). These findings suggest that our subtype classification method has the advantage of distinguishing LAML cases within the same subtypes identified by other methods, as they exhibit significantly different clinical outcomes.

## Discussion

**FIGURE 8:**
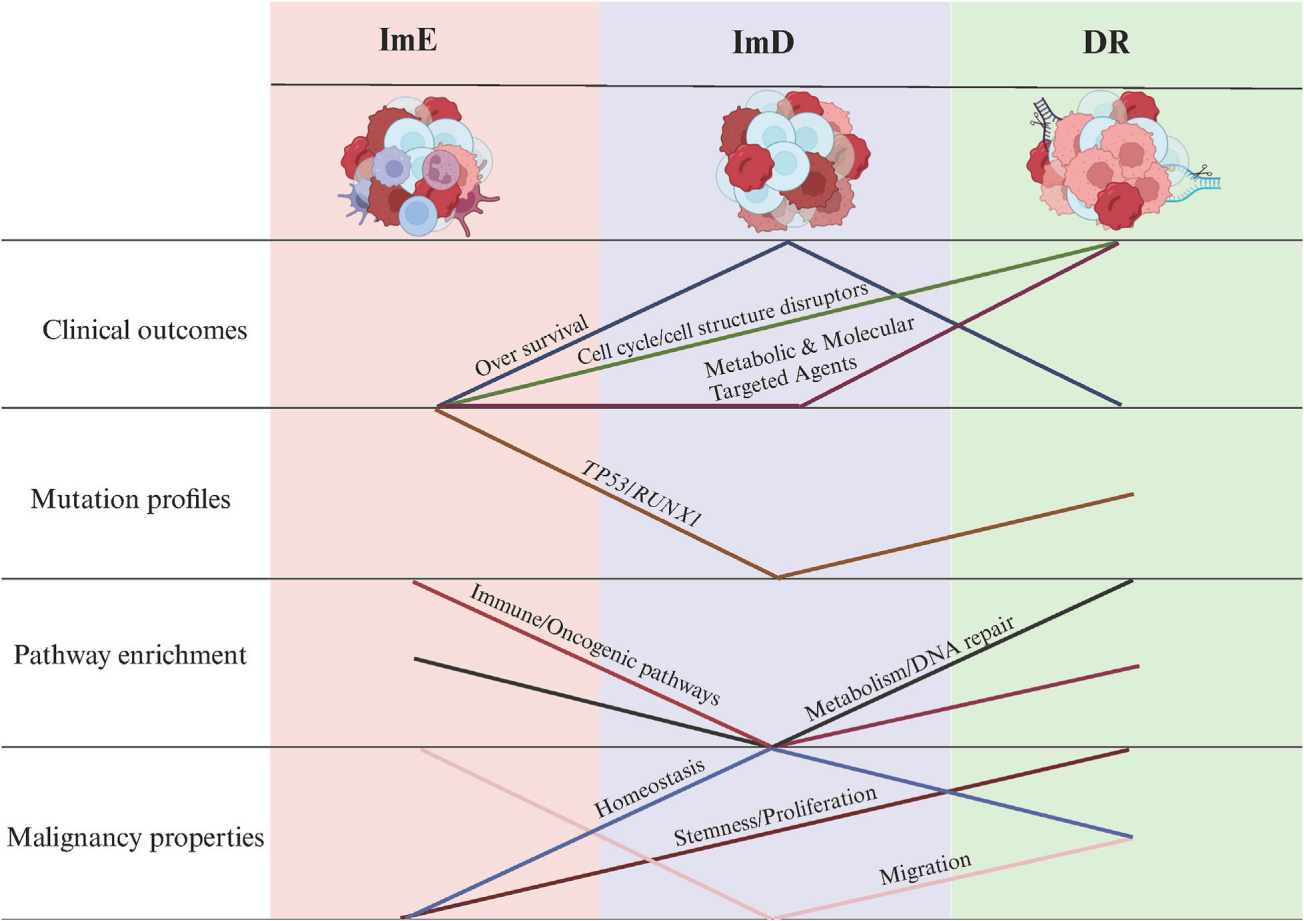
Summary of the clinical and molecular characteristics of the AML subtypes. The figure was created with BioRender.com.

Uncovering cancer subtypes is a crucial strategy for precision oncology. For the first time, we identified AML subtypes based on pathway module enrichment. Using 14 pathways related to metabolism, immune, DNA repair, and oncogenic signaling, we revealed three AML subtypes: DR, ImD, and ImE, consistently in four independent cohorts. DR is marked by high expressions of DNA repair and metabolic pathways, high stemness and proliferation potential, as well as high sensitivity to cell cycle/cell structure disruptors. ImD is characterized by low expressions of immune and oncogenic pathways, favorable survival prognosis, low mutation rates of *RUNX1* and *TP53*, high homeostasis, and low migration potential. ImE exhibits high enrichment of immune and oncogenic pathways, low stemness and proliferation capacity, low homeostasis, high migration potential, and low sensitivity to all agents (Figure 8). The lack of significant prognostic differences among pediatric AML subtypes, despite clustering patterns resembling those of adult AML, may be due to multiple factors. Biologically, pediatric AML exhibits distinct driver mutation profiles (e.g., fewer TP53 mutations, more KMT2A rearrangements) (Bolouri et al., 2018) and epigenetic landscapes (Xu et al., 2022) that alter the clinical impact of shared pathway activations. Treatment differences also play a role: children tolerate intensive chemotherapy better (de Rooij et al., 2015), potentially masking subtype-specific outcomes, while adult-targeted therapies (e.g., FLT3 inhibitors) (Döhner et al., 2021) amplify prognostic disparities. These findings highlight the need for pediatric-specific risk stratification integrating developmental and therapeutic contexts. Different from many solid tumors in which low enrichment of immune signatures is associated with worse clinical outcomes, such as breast cancer (He et al., 2018) and melanoma (Liu et al., 2021), the low enrichment of immune pathways is a positive prognostic factor in AML, as evidenced by the immune-deprived subtype (ImD) showing the highest overall survival rates. It is reasonable since AML is a malignancy caused by uncontrolled clonal proliferation of immune cells (myeloid progenitor cells) (Perzolli et al., 2024). In AML, the malignant cells are themselves immune-derived, and heightened activity in immune-related pathways—such as T cell receptor signaling or JAK-STAT signaling—may reflect increased leukemic cell activity or aggressiveness. Conversely, the ImD subtype’s low enrichment of these pathways, coupled with reduced stemness, proliferation, and migration potential (Figure 4), suggests a less aggressive disease state, contributing to its favorable prognosis. This interpretation is consistent with emerging evidence that dysregulated immune signaling can promote AML progression (Perzolli et al., 2024), highlighting the unique prognostic implications of immune pathway activity in this hematologic malignancy.

Our AML subtyping method has significant clinical implications. First, this AML classification has prognostic value, as the ImD subtype exhibits a more favorable prognosis than the other subtypes. Second, our AML classification may guide clinical treatment. Our mechanistic stratification of chemotherapeutic agents revealed subtype-specific vulnerabilities: The DR subtype displayed the highest sensitivity to cell cycle/structural disruptors (e.g., methotrexate, SB.743921; *P* < 0.001 vs ImE), followed by the ImD subtype, whereas the ImE subtype exhibited consistently poor responsiveness (Figure 5), potentially due to enhanced DNA repair capacity. In contrast, for metabolic and molecular targeted agents (e.g., daporinad, decitabine), sensitivity patterns diverged: DR and ImD subtypes showed comparable responses (*P* > 0.05), while ImE maintained the lowest sensitivity across all agents (*P* < 0.01; Figure 8). This outcome suggests that while DR malignancies retain dependence on proliferative pathways, ImE tumors may employ broad resistance mechanisms, such as metabolic plasticity to bypass NAD depletion or epigenetic reservoir stability to counteract DNMT inhibition. These findings underscore the need for subtype-specific therapeutic strategies, prioritizing cell cycle disruptors for DR subtype patients and combinatorial approaches targeting non-proliferative vulnerabilities in ImE subtype malignancies. For example, the activation of DNA repair pathways suggests that the AML cells have enhanced DNA repair capabilities, making them resistant to conventional chemotherapy. Thus, targeted therapies that disrupt DNA repair mechanisms, such as PARP inhibitors (Padella et al., 2022), could be more effective against the DR subtype of AML patients. Also, targeting the cell cycle pathway, such as CDK4/6 inhibitors (Abbas et al., 2021), may be effective for this subtype of AML patients. For the ImE subtype of patients, inhibitors of oncogenic pathways, such as PI3K/Akt, TGF-*β*, Wnt, Hedgehog, and mTOR, could be relatively effective.

While our study provides valuable insights into the molecular and clinical characteristics of AML subtypes, there are several limitations that need to be addressed. First, the datasets used in this study are primarily from adult patients, and further validation in pediatric cohorts is necessary to ensure the generalizability of our findings. Second, translating these molecular insights into improved patient outcomes and treatment would require rigorous experimental and clinical validation. Lastly, while our subtyping framework identifies clinically actionable patterns, translating these insights into targeted therapies will require functional validation in preclinical models.

In conclusion, our pathway enrichment-based subtyping approach offers a promising framework for understanding the molecular heterogeneity of AML and guiding personalized treatment strategies. By identifying subtypes with distinct pathway activities, molecular features, immune profiles, clinical outcomes, and drug sensitivities, we aim to improve the clinical management of AML.

## Data Availability

The original contributions presented in the study are included in the article/Supplementary Material, further inquiries can be directed to the corresponding authors.

## References

[B1] Abbas H. A. Alaniz Z. Mackay S. Cyr M. Zhou J. Issa G. C. (2021). Single-cell polyfunctional proteomics of CD4 cells from patients with AML predicts responses to anti-PD-1-based therapy. Blood Adv. 5, 4569–4574. 10.1182/bloodadvances.2021004583 34555853 PMC8759127

[B2] AbbVie Inc. and Genentech Inc. (2020). VENCLEXTA® (venetoclax): US prescribing informationNorth Chicago, IL, USA & South San Francisco, CA, USA. Available online at: https://www.accessdata.fda.gov/drugsatfda_docs/label/2020/208573s020s021lbl.pdf (Accessed October 16, 2020).

[B3] Agios Pharmaceuticals (2018). TIBSOVO® (ivosidenib). US prescribing Information. Available online at: https://www.accessdata.fda.gov/drugsatfda_docs/label/2018/211192s000lbl.pdf (Accessed August 11, 2018).

[B4] Aplenc R. Alonzo T. A. Gerbing R. B. Lange B. J. Hurwitz C. A. Wells R. J. (2008). Safety and efficacy of gemtuzumab ozogamicin in combination with chemotherapy for pediatric acute myeloid leukemia: a report from the children's oncology group. J. Clin. Oncol. 26, 2390–3295. 10.1200/JCO.2007.13.0096 18467731 PMC4558626

[B5] Aplenc R. Meshinchi S. Sung L. Alonzo T. Choi J. Fisher B. (2020). Bortezomib with standard chemotherapy for children with acute myeloid leukemia does not improve treatment outcomes: a report from the Children's Oncology Group. Haematologica 105, 1879–1886. 10.3324/haematol.2019.220962 32029509 PMC7327649

[B6] Appelbaum F. R. Bernstein I. D. (2017). Gemtuzumab ozogamicin for acute myeloid leukemia. Blood 130, 2373–2376. 10.1182/blood-2017-09-797712 29021230

[B7] Arber D. A. Orazi A. Hasserjian R. Thiele J. Borowitz M. J. Le Beau M. M. (2016). The 2016 revision to the World Health Organization classification of myeloid neoplasms and acute leukemia. Blood 127, 2391–2405. 10.1182/blood-2016-03-643544 27069254

[B8] Astellas Pharma (2018). XOSPATA® (gilteritinib). US Prescribing Information. Available online at: https://www.accessdata.fda.gov/drugsatfda_docs/label/2018/211349s000lbl.pdf (Accessed December 24, 2018).

[B9] Benjamini Y. Hochberg Y. (1995). Controlling the false discovery rate: a practical and powerful approach to multiple testing. J. R. Stat. Soc. Ser. B Methodol. 57, 289–300. 10.1111/j.2517-6161.1995.tb02031.x

[B10] Bennett J. M. Catovsky D. Daniel M. T. Flandrin G. Galton D. A. Gralnick H. R. (1976). Proposals for the classification of the acute leukaemias. French-American-British (FAB) co-operative group. Br. J. Haematol. 33, 451–458. 10.1111/j.1365-2141.1976.tb03563.x 188440

[B11] Bland J. M. Altman D. G. (1998). Survival probabilities (the Kaplan-Meier method). BMJ 317, 1572. 10.1136/bmj.317.7172.1572 9836663 PMC1114388

[B12] Bolouri H. Farrar J. E. Triche T. Jr Ries R. E. Lim E. L. Alonzo T. A. (2018). The molecular landscape of pediatric acute myeloid leukemia reveals recurrent structural alterations and age-specific mutational interactions. Nat. Med. 24, 103–112. 10.1038/nm.4439 29227476 PMC5907936

[B13] Carter J. L. Hege K. Yang J. Kalpage H. A. Su Y. Edwards H. (2020). Targeting multiple signaling pathways: the new approach to acute myeloid leukemia therapy. Signal Transduct. Target. Ther. 5, 288. 10.1038/s41392-020-00361-x 33335095 PMC7746731

[B14] Celgene Corporation (2017). IDHIFA® (enasidenib) tablets: US prescribing information. Available online at: https://www.accessdata.fda.gov/drugsatfda_docs/label/2017/209606s000lbl.pdf (Accessed August 30, 2017).

[B15] Chuang M.-K. Chiu Y. C. Chou W. C. Hou H. A. Tseng M. H. Kuo Y. Y. (2015). An mRNA expression signature for prognostication in *de novo* acute myeloid leukemia patients with normal karyotype. Oncotarget 6 (36), 39098–39110. 10.18632/oncotarget.5390 26517675 PMC4770759

[B16] Darici S. Alkhaldi H. Horne G. Jørgensen H. G. Marmiroli S. Huang X. (2020). Targeting PI3K/Akt/mTOR in AML: rationale and clinical evidence. J. Clin. Med. 9, 2934. 10.3390/jcm9092934 32932888 PMC7563273

[B17] de Rooij J. D. Zwaan C. M. van den Heuvel-Eibrink M. (2015). Pediatric AML: from biology to clinical management. J. Clin. Med. 4, 127–149. 10.3390/jcm4010127 26237023 PMC4470244

[B18] Döhner H. Estey E. Grimwade D. Amadori S. Appelbaum F. R. Büchner T. (2017). Diagnosis and management of AML in adults: 2017 ELN recommendations from an international expert panel. Blood 129, 424–447. 10.1182/blood-2016-08-733196 27895058 PMC5291965

[B19] Döhner H. Wei A. H. Appelbaum F. R. Craddock C. DiNardo C. D. Dombret H. (2022). Diagnosis and management of AML in adults: 2022 recommendations from an international expert panel on behalf of the ELN. Blood 140, 1345–1377. 10.1182/blood.2022016867 35797463

[B20] Döhner H. Wei A. H. Löwenberg B. (2021). Towards precision medicine for AML. Nat. Rev. Clin. Oncol. 18, 577–590. 10.1038/s41571-021-00509-w 34006997

[B21] Döhner H. Weisdorf D. J. Bloomfield C. D. (2015). Acute myeloid leukemia. 373, 1136–1152. 10.1056/NEJMra1406184 26376137

[B22] Dong M. Blobe G. C. (2006). Role of transforming growth factor-beta in hematologic malignancies. Blood 107, 4589–4596. 10.1182/blood-2005-10-4169 16484590 PMC1895802

[B23] Eleni L. D. Nicholas Z. C. Alexandros S. (2010). Challenges in treating older patients with acute myeloid leukemia. J. Oncol. 2010, 943823. 10.1155/2010/943823 20628485 PMC2902223

[B24] Feldman E. J. Lancet J. E. Kolitz J. E. Ritchie E. K. Roboz G. J. List A. F. (2011). First-in-man study of CPX-351: a liposomal carrier containing cytarabine and daunorubicin in a fixed 5:1 molar ratio for the treatment of relapsed and refractory acute myeloid leukemia. J. Clin. Oncol. official J. Am. Soc. Clin. Oncol. 29, 979–985. 10.1200/JCO.2010.30.5961 PMC4520927 21282541

[B25] Gamis A. S. Alonzo T. A. Meshinchi S. Sung L. Gerbing R. B. Raimondi S. C. (2014). Gemtuzumab ozogamicin in children and adolescents with *de novo* acute myeloid leukemia improves event-free survival by reducing relapse risk: results from the randomized phase III children's oncology group trial AAML0531. J. Clin. Oncol. 32, 3021–3032. 10.1200/JCO.2014.55.3628 25092781 PMC4162498

[B26] Guo S. Mohan G. S. Wang B. Li T. Daver N. Zhao Y. (2024). Paired single-B-cell transcriptomics and receptor sequencing reveal activation states and clonal signatures that characterize B cells in acute myeloid leukemia. J. Immunother. cancer 12, e008318. 10.1136/jitc-2023-008318 38418394 PMC10910691

[B27] Hänzelmann S. Castelo R. Guinney J. (2013). GSVA: gene set variation analysis for microarray and RNA-Seq data. BMC Bioinforma. 14, 7. 10.1186/1471-2105-14-7 PMC3618321 23323831

[B28] Hassan C. Afshinnekoo E. Li S. Wu S. Mason C. E. (2017). Genetic and epigenetic heterogeneity and the impact on cancer relapse. Exp. Hematol. 54, 26–30. 10.1016/j.exphem.2017.07.002 28705639 PMC5651672

[B29] He Y. Jiang Z. Chen C. Wang X. (2018). Classification of triple-negative breast cancers based on Immunogenomic profiling. J. Exp. Clin. Cancer Res. 37, 327. 10.1186/s13046-018-1002-1 30594216 PMC6310928

[B30] Heuser M. Ofran Y. Boissel N. Brunet Mauri S. Craddock C. Janssen J. (2020). Acute myeloid leukaemia in adult patients: ESMO Clinical Practice Guidelines for diagnosis, treatment and follow-up. Ann. Oncol. 31, 697–712. 10.1016/j.annonc.2020.02.018 32171751

[B31] Hoy S. M. (2019). Glasdegib: first global approval. Drugs 79, 207–213. 10.1007/s40265-018-1047-7 30666593

[B32] Khoury J. D. Solary E. Abla O. Akkari Y. Alaggio R. Apperley J. F. (2022). The 5th edition of the World Health organization classification of haematolymphoid tumours: myeloid and histiocytic/dendritic neoplasms. Leukemia 36, 1703–1719. 10.1038/s41375-022-01613-1 35732831 PMC9252913

[B33] Láinez-González D. Alonso-Aguado A. B. Alonso-Dominguez J. M. (2023). Understanding the Wnt signaling pathway in acute myeloid leukemia stem cells: a feasible key against relapses. Biology 12, 683. 10.3390/biology12050683 37237497 PMC10215262

[B34] Langfelder P. Horvath S. (2008). WGCNA: an R package for weighted correlation network analysis. BMC Bioinforma. 9, 559. 10.1186/1471-2105-9-559 PMC2631488 19114008

[B35] Lei J. L. Luo J. T. Liu Q. Wang X. S. (2024). Identifying cancer subtypes based on embryonic and hematopoietic stem cell signatures in pan-cancer. Cell Oncol. 47, 587–605. 10.1007/s13402-023-00886-7 PMC12973968 37821797

[B36] Ley T. J. Miller C. Ding L. Raphael B. J. Mungall A. J. (2013). Genomic and epigenomic landscapes of adult *de novo* acute myeloid leukemia. N. Engl. J. Med. 368, 2059–2074. 10.1056/NEJMoa1301689 23634996 PMC3767041

[B37] Liu Q. Nie R. Li M. Li L. Zhou H. Lu H. (2021). Identification of subtypes correlated with tumor immunity and immunotherapy in cutaneous melanoma. Comput. Struct. Biotec 19, 4472–4485. 10.1016/j.csbj.2021.08.005 PMC8379294 34471493

[B38] Lohse I. Statz-Geary K. Brothers S. P. Wahlestedt C. (2018). Precision medicine in the treatment stratification of AML patients: challenges and progress. Oncotarget 9, 37790–37797. 10.18632/oncotarget.26492 30701032 PMC6340870

[B39] Maeser D. Gruener R. F. Huang R. S. (2021). oncoPredict: an R package for predicting *in vivo* or cancer patient drug response and biomarkers from cell line screening data. Briefings Bioinforma. 22, bbab260. 10.1093/bib/bbab260 PMC8574972 34260682

[B40] Mayakonda A. Lin D. C. Assenov Y. Plass C. Koeffler H. P. (2018). Maftools: efficient and comprehensive analysis of somatic variants in cancer. Genome Res. 28, 1747–1756. 10.1101/gr.239244.118 30341162 PMC6211645

[B41] McInnes L. Healy J. Saul N. Umap L. J. J. O. S. S. (2018). UMAP: uniform manifold approximation and projection. J. Open Source Softw. 3, 861. 10.21105/joss.00861

[B42] Merino A. Maakaron J. Bachanova V. (2023). Advances in NK cell therapy for hematologic malignancies: NK source, persistence and tumor targeting. Blood Rev. 60, 101073. 10.1016/j.blre.2023.101073 36959057 PMC10979648

[B43] Moser B. Edtmayer S. Witalisz-Siepracka A. Stoiber D. (2021). The ups and downs of STAT inhibition in acute myeloid leukemia. Biomedicines 9, 1051. 10.3390/biomedicines9081051 34440253 PMC8392322

[B44] Naji N. S. Sathish M. Karantanos T. (2024). Inflammation and related signaling pathways in acute myeloid leukemia. Cancers (Basel) 16, 3974. 10.3390/cancers16233974 39682161 PMC11640130

[B45] Nepstad I. Hatfield K. J. Grønningsæter I. S. Reikvam H. (2020). The PI3K-Akt-mTOR signaling pathway in human acute myeloid leukemia (AML) cells. Int. J. Mol. Sci. 21, 2907. 10.3390/ijms21082907 32326335 PMC7215987

[B46] Novartis Pharmaceuticals Corp (2017). RYDAPT® (midostaurin) capsules: US prescribing information. Available online at: https://www.accessdata.fda.gov/drugsatfda_docs/label/2017/207997s000lbl.pdf (Accessed May 16, 2017).

[B47] null n. Ley T. J. Miller C. Ding L. Raphael B. J. Mungall A. J. (2013). Genomic and epigenomic landscapes of adult *de novo* acute myeloid leukemia. N. Engl. J. Med. 368, 2059–2074. 10.1056/NEJMoa1301689 23634996 PMC3767041

[B48] Padella A. Ghelli Luserna Di Rorà A. Marconi G. Ghetti M. Martinelli G. Simonetti G. (2022). Targeting PARP proteins in acute leukemia: DNA damage response inhibition and therapeutic strategies. J. Hematol. & Oncol. 15, 10. 10.1186/s13045-022-01228-0 35065680 PMC8783444

[B49] Papaemmanuil E. Gerstung M. Bullinger L. Gaidzik V. I. Paschka P. Roberts N. D. (2016a). Genomic classification and prognosis in acute myeloid leukemia. N. Engl. J. Med. 374, 2209–2221. 10.1056/NEJMoa1516192 27276561 PMC4979995

[B50] Papaemmanuil E. Gerstung M. Bullinger L. Gaidzik V. I. Paschka P. Roberts N. D. (2016b). Genomic classification and prognosis in acute myeloid leukemia. N. Engl. J. Med. 374, 2209–2221. 10.1056/NEJMoa1516192 27276561 PMC4979995

[B51] Perzolli A. Koedijk J. B. Zwaan C. M. Heidenreich O. (2024). Targeting the innate immune system in pediatric and adult AML. Leukemia 38, 1191–1201. 10.1038/s41375-024-02217-7 38459166 PMC11147779

[B52] Pfizer (2018). DaurismoTM (glasdegib) tablets, for oral use: US prescribing information. Available online at: https://www.accessdata.fda.gov/drugsatfda_docs/label/2018/210656s000lbl.pdf (Accessed November 26, 2018).

[B53] Sanchez-Vega F. Mina M. Armenia J. Chatila W. K. Luna A. La K. C. (2018). Oncogenic signaling pathways in the cancer genome Atlas. Cell 173, 321–337.e10. 10.1016/j.cell.2018.03.035 29625050 PMC6070353

[B54] Shallis R. M. Bewersdorf J. P. Boddu P. C. Zeidan A. M. (2019). Hedgehog pathway inhibition as a therapeutic target in acute myeloid leukemia. Expert Rev. Anticancer Ther. 19, 717–729. 10.1080/14737140.2019.1652095 31422721

[B55] Shannon P. Markiel A. Ozier O. Baliga N. S. Wang J. T. Ramage D. (2003). Cytoscape: a software environment for integrated models of biomolecular interaction networks. Genome Res. 13, 2498–2504. 10.1101/gr.1239303 14597658 PMC403769

[B56] Szklarczyk D. Gable A. L. Lyon D. Junge A. Wyder S. Huerta-Cepas J. (2019). STRING v11: protein–protein association networks with increased coverage, supporting functional discovery in genome-wide experimental datasets. Nucleic Acids Res. 47, D607-D613–D613. 10.1093/nar/gky1131 30476243 PMC6323986

[B57] Terao T. Minami Y. (2019). Targeting Hedgehog (hh) pathway for the acute myeloid leukemia treatment. Cells 8, 312. 10.3390/cells8040312 30987263 PMC6523210

[B58] Tobias H. Jurinovic V. Batcha A. M. N. Bamopoulos S. A. Rothenberg-Thurley M. Ksienzyk B. (2018). A 29-gene and cytogenetic score for the prediction of resistance to induction treatment in acute myeloid leukemia. Haematologica 103, 456–465. 10.3324/haematol.2017.178442 29242298 PMC5830382

[B59] Tyner J. W. Tognon C. E. Bottomly D. Wilmot B. Kurtz S. E. Savage S. L. (2018). Functional genomic landscape of acute myeloid leukaemia. Nature 562, 526–531. 10.1038/s41586-018-0623-z 30333627 PMC6280667

[B60] van Galen P. Hovestadt V. Wadsworth Ii M. H. Hughes T. K. Griffin G. K. Battaglia S. (2019). Single-cell RNA-seq reveals AML hierarchies relevant to disease progression and immunity. Cell 176, 1265–1281. 10.1016/j.cell.2019.01.031 30827681 PMC6515904

[B61] Venugopal S. Bar-Natan M. Mascarenhas J. O. (2020). JAKs to STATs: a tantalizing therapeutic target in acute myeloid leukemia. Blood Rev. 40, 100634. 10.1016/j.blre.2019.100634 31677846

[B62] Wang C. Y. Lin S. C. Chang K. J. Cheong H. P. Wu S. R. Lee C. H. (2024). Immunoediting in acute myeloid leukemia: reappraising T cell exhaustion and the aberrant antigen processing machinery in leukemogenesis. Heliyon 10, e39731. 10.1016/j.heliyon.2024.e39731 39568858 PMC11577197

[B63] Xu H. Wen Y. Jin R. Chen H. (2022). Epigenetic modifications and targeted therapy in pediatric acute myeloid leukemia. Front. Pediatr. 10, 975819. 10.3389/fped.2022.975819 36147798 PMC9485478

[B64] Yang Y. Pu J. Yang Y. (2024). Glycolysis and chemoresistance in acute myeloid leukemia. Heliyon 10, e35721. 10.1016/j.heliyon.2024.e35721 39170140 PMC11336864

