# Supplementary figures and images for: Identifying acute myeloid leukemia subtypes based on pathway enrichment
Source: Front Pharmacol. 2025 Mar 21;16:1557112. doi: 10.3389/fphar.2025.1557112 (PMC11968745; doi:10.3389/fphar.2025.1557112)

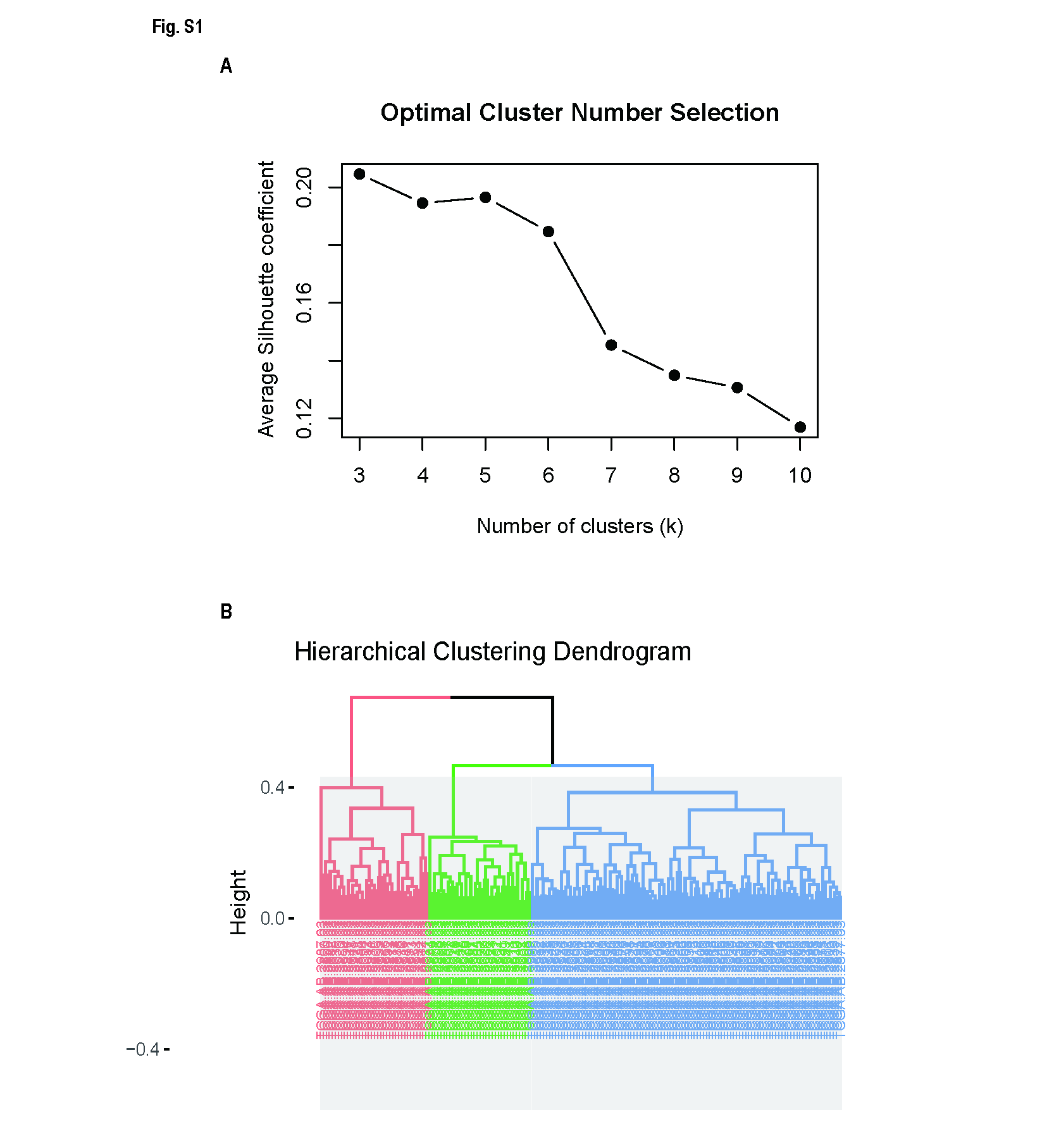

Supplement: Supplementary file 2 [file Image1.TIFF]

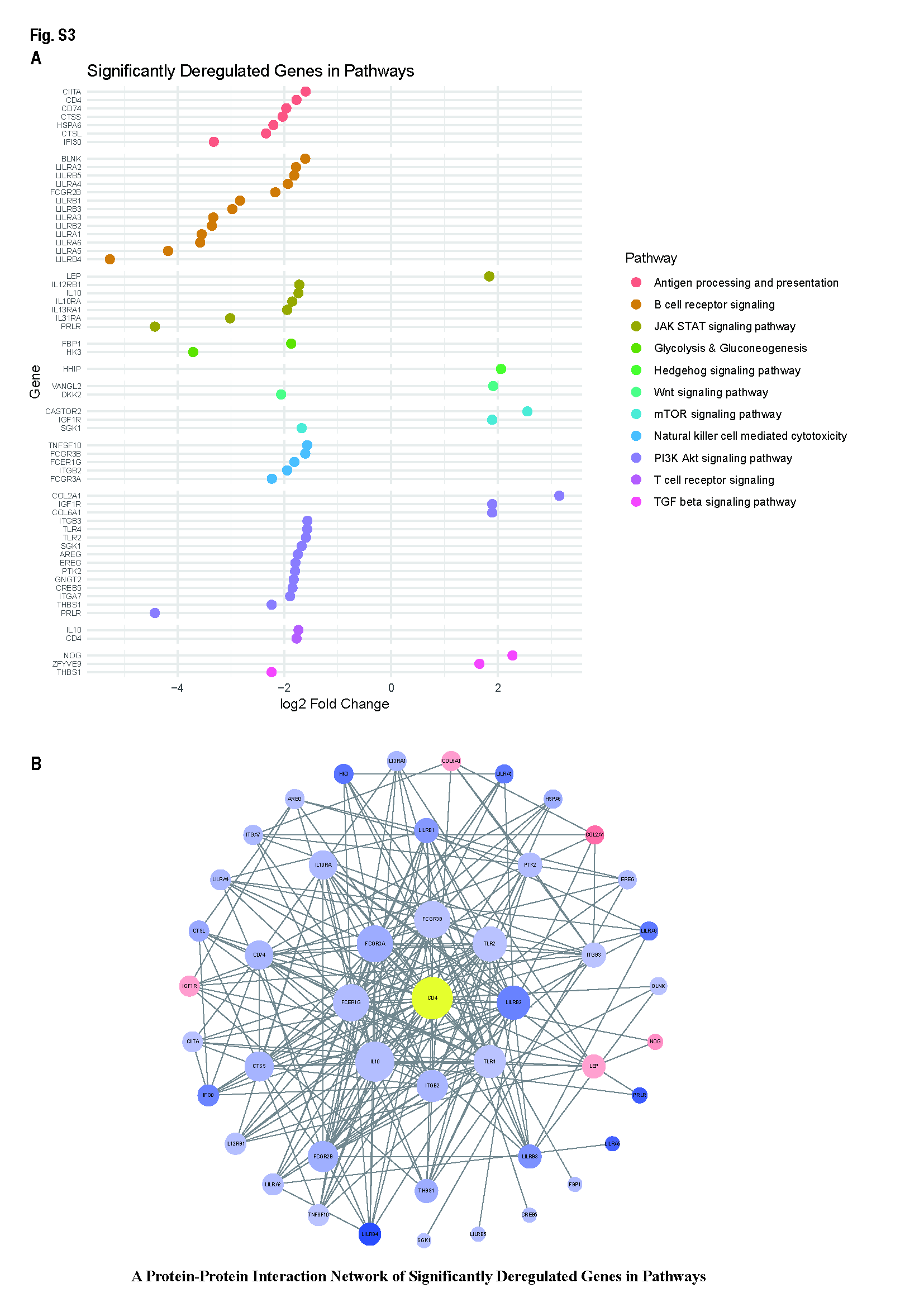

Supplement: Supplementary file 4 [file Image3.TIF]

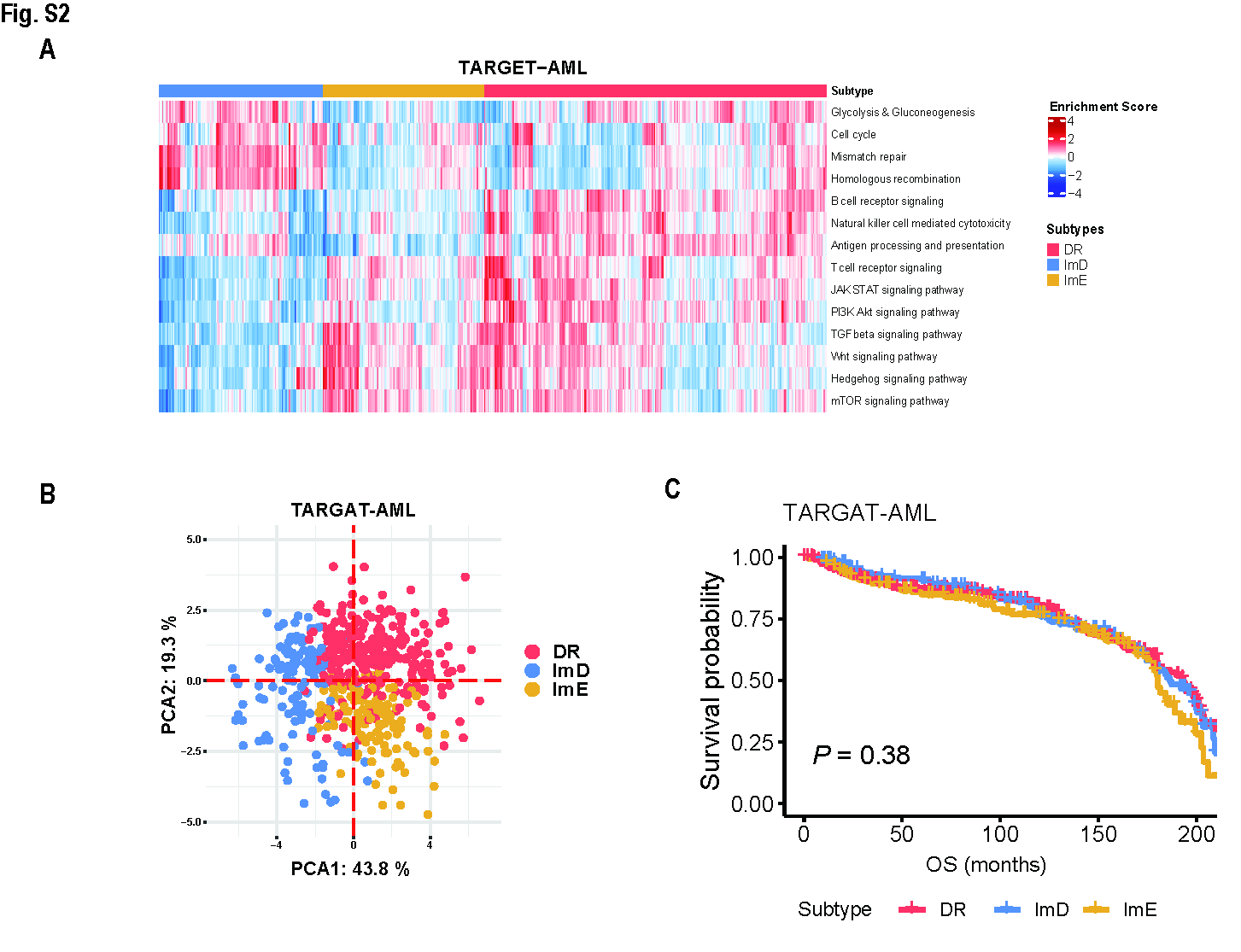

Supplement: Supplementary file 7 [file Image2.TIFF]
